# Combined Ultrasound and Cone Beam CT Improves Target Segmentation for Image Guided Radiation Therapy in Uterine Cervix Cancer

**DOI:** 10.1016/j.ijrobp.2019.03.003

**Published:** 2019-07-01

**Authors:** Sarah A. Mason, Ingrid M. White, Tuathan O'Shea, Helen A. McNair, Sophie Alexander, Eleftheria Kalaitzaki, Jeffrey C. Bamber, Emma J. Harris, Susan Lalondrelle

**Affiliations:** ∗ Institute of Cancer Research, Radiotherapy and Imaging, London, United Kingdom; † Radiotherapy Department, Royal Marsden NHS Foundation Trust, London, United Kingdom; ‡ Radiotherapy Physics Department, Royal Marsden NHS Foundation Trust, London, United Kingdom; § Clinical Trials Unit, Royal Marsden NHS Foundation Trust, London, United Kingdom

## Abstract

**Purpose:**

Adaptive radiation therapy strategies could account for interfractional uterine motion observed in patients with cervix cancer, but the current cone beam computed tomography (CBCT)-based treatment workflow is limited by poor soft-tissue contrast. The goal of the present study was to determine if ultrasound (US) could be used to improve visualization of the uterus, either as a single modality or in combination with CBCT.

**Methods and Materials:**

Interobserver uterine contour agreement and confidence were compared on 40 corresponding CBCT, US, and CBCT-US-fused images from 11 patients with cervix cancer. Contour agreement was measured using the Dice similarity coefficient (DSC) and mean contour-to-contour distance (MCCD). Observers rated their contour confidence on a scale from 1 to 10. Pairwise Wilcoxon signed-rank tests were used to measure differences in contour agreement and confidence.

**Results:**

CBCT-US fused images had significantly better contour agreement and confidence than either individual modality (*P* < .05), with median (interquartile range [IQR]) values of 0.84 (0.11), 1.26 (0.23) mm, and 7 (2) for the DSC, MCCD, and observer confidence ratings, respectively. Contour agreement was similar between US and CBCT, with median (IQR) DSCs of 0.81 (0.17) and 0.82 (0.14) and MCCDs of 1.75 (1.15) mm and 1.62 (0.74) mm. Observers were significantly more confident in their US-based contours than in their CBCT-based contours (*P* < .05), with median (IQR) confidence ratings of 7 (2.75) versus 5 (4).

**Conclusions:**

CBCT and US are complementary and improve uterine segmentation precision when combined. Observers could localize the uterus with a similar precision on independent US and CBCT images.

**Summary**

Ultrasound (US) and cone beam computed tomography (CBCT) were assessed (1) as independent modalities and (2) in combination for localizing the uterus in 11 patients with cervix cancer for adaptive radiation therapy purposes. Interobserver uterine contour agreement was similar on independent US and CBCT images, but observers were significantly more confident in their US-based contours. However, both interobserver contour agreement and contour confidence were significantly improved when US and CBCT were combined; the 2 modalities were shown to be complementary.

## Introduction

The primary clinical target volume (CTV_p_) in cervix radiation therapy (RT) includes the uterus and cervix,^1^ which are highly mobile structures. Interfraction motion ranging from 2 to 60 mm has been observed as a result of changes in bladder and rectal volume and tumor regression.^2^, ^3^, ^4^, ^5^ Generous CTV_p_ to planning target volume margins are necessary to compensate for positional uncertainty, but this increases the volume of normal tissue within the prescription dose region, which increases the risk of toxicity.^6^, ^7^, ^8^, ^9^, ^10^

Cone beam computed tomography (CBCT) is now widely available for bone, fiducial,^11^, ^12^ and soft tissue-based treatment verification^12^, ^13^, ^14^, ^15^, ^16^, ^17^, ^18^ and plan of the day selection.^12^, ^19^ Disadvantages of CBCT include additional radiation dose and poor soft-tissue contrast because of scatter and reconstruction artifacts. Indeed, image quality on 12% to 18% of gynecological and prostate CBCT has been reported to be too poor for soft-tissue visualization purposes.^13^, ^14^, ^15^, ^16^, ^17^, ^18^ Even on CBCT scans deemed suitable for soft-tissue analyses, poor image quality is reported to be the source of high interobserver contouring variability of the uterus and prostate.^15^, ^17^

Ultrasound (US) could be an effective, nonionizing, low-cost solution for providing high-quality images of the pelvic anatomy at the time of radiation treatment delivery. US is routinely used in gynecologic/obstetric applications and could be a promising alternative or adjunct to CBCT for image guidance in cervix cancer RT.^11^, ^20^, ^21^ Baker et al observed the apparent superiority of US image quality compared with CBCT in visualizing the uterus in a study investigating interfractional uterine motion, but no quantitative analysis was performed.^11^ However, there are some drawbacks associated with US imaging of the uterus and cervix. (Note: for the remainder of this text, the uterus and cervix complex will be considered a single structure and will be referred to as the uterus). US image quality is operator dependent and varies depending on the amount of abdominal fat, bladder volume, amount of probe pressure applied, and presence of obstructions such as gas or bone in the beam path.^22^, ^23^, ^24^, ^25^ Additionally, mechanically swept 3-dimensional (3D) probes have a relatively small field of view, making it difficult to capture the entire uterus (particularly in cases where the disease is bulky) and impossible to capture all of the nearby organs at risk, particularly the rectum and bowel and involved lymph nodes, within a single sweep.

A quantitative comparison of uterine segmentation on US and CBCT has not been evaluated previously. We expected interpatient variability in uterus visualization and that either modality may be superior depending on patient characteristics at the time of treatment. It is not known if combining/fusing CBCT and US provides additional benefit in accuracy and precision of uterus localization for soft tissue–based treatment verification, plan of the day selection, and online replanning.

The purpose of this work was to compare interobserver agreement in uterine segmentation and observer confidence in segmentation on CBCT, US, and CBCT-US fused images to determine the optimal imaging method for target localization during cervix RT.

## Methods and Materials

### Patients and treatment

Eleven patients with biopsy-proven diagnosis of locally advanced cervix cancer were included in this National Health Services Research Ethics–approved study (reference: 15/LO/1438). Fédération Internationale de Gynécologie Obstétrique stage distribution was as follows: IIA = 1, IIB = 9, IIIB = 0, IVA = 1. The mean patient age was 51 (±16) years. Patients were treated with radical chemoradiotherapy from February 2016 to May 2017. Patients were instructed to drink 350 mL water after complete bladder voiding 1 hour before treatment as per institutional protocol to maintain interfractional bladder volume and consistent setup. There were no bowel preparation instructions. Kilovoltage CBCT images were acquired immediately before treatment for online correction based on bony registration on days 1 to 3 and weekly thereafter unless there was a systematic error of >5 mm or clinical indication, in which case they were acquired more frequently. 3D US of the uterus were acquired using the Clarity system (Elekta Ltd, Stockholm, Sweden) immediately before CBCT acquisition at 4 to 6 treatment sessions after patient setup on the RT treatment couch.

### Data acquisition

#### US imaging

3D US scans (5 MHz center frequency, mechanically swept probe) were acquired using the Clarity system. The Clarity system is described in detail elsewhere,^26^ but briefly, it is a standard US imaging system that is integrated into the RT clinic via infrared tracking, whereby the position of the probe (and the corresponding US images) with respect to the isocenter of the treatment room is known with submillimeter accuracy. US operators (either a clinical oncologist or a therapeutic radiographer) applied a thick layer of US gel to the probe and scanned the uterus transabdominally using the smallest probe pressure possible to minimize soft-tissue deformation while still obtaining clear visualization of the uterus.

#### CBCT imaging

CBCT imaging (Elekta Ltd) was performed immediately after US scanning, with no more than a 5-minute interval between US and CBCT scans. CBCT imaging parameters were 120 kVp and 80 mAs with 350 projections and a bowtie filter.

Sixty-four US-CBCT image pairs (128 images in total) were obtained as part of this study. Two image pairs were excluded because of US operator errors in the probe calibration step of the Clarity QA, which caused misregistration of US images to the treatment room isocenter. Five image pairs were excluded because of failure to save US or CBCT scans. Of the 57 remaining image pairs, 40 were randomly selected for analysis, leaving the remainder (17 image pairs) exclusively for observer training purposes.

### Image formatting

#### Image registration

CBCT images were registered to the planning computed tomography (CT) scan using the Synergy bone match algorithm. Translational and rotational error was summarized as a translational couch shift. After export to the Clarity workstation, these translational shifts were applied to each CBCT scan to replicate match to planning CT. The infrared tracking technology provided by the Clarity system enabled spatial registration between the US images and the CBCT images. In the offline Clarity workstation, the same translational moves were applied to the corresponding US images.

#### Image presentation

A software application was written in Matlab (MathWorks, Natick, MA) to enable presentation of CT-CBCT, CT-US, and CBCT-US registration for assessment by observers. The pixel size (in the format of [superoinferior direction, anteroposterior direction]) was [2.5, 1] mm for CBCT images, [0.58, 0.58] mm for US, and [3, 1] mm for CT. Registered images were superimposed over one another, with user-adjustable sliders for 3D slice selection, transparency, and windowing in both the sagittal and axial orientations. This functionality was achieved by interpolating the image in the registration with the larger pixels to a grid matching the sampling density of the image with the smaller pixels. Note that any references to “CBCT” or “US” hereafter imply registration with the planning CT, as this is standard clinical practice. In the case of CBCT-US registration (henceforth referred to as CBCT-US fusion), the planning CT was available for reference in a separate window rather than as a third superimposed image.

### Image contouring and rating

Observers used the MATLAB application for all image analysis, including

1. Delineation of the uterus on a pr-selected 2D sagittal slice: The sagittal slice used for contouring was the centermost slice of the uterus, as identified by one of the observers in the Experienced cohort (described in next section). This slice was in the same position for corresponding CBCT, US, and CBCT-US fusion images.
2. Contour confidence ratings: After contouring the uterus, each observer was asked to rate the confidence that his or her contour reflected the true uterine boundary on a scale from 1 (extremely unconfident) to 10 (extremely confident).

Observers first evaluated CBCT and US images separately. These images were displayed in a random order so that observers were blind to which CBCT and US images were paired and which images were from the same patient. Observers then evaluated the CBCT-US fusion images, which were also displayed in a random order.

One experienced observer also contoured the bladder on the central 2-dimensional sagittal slice on US and CBCT so that the relationship between bladder size and image quality could be assessed.

### Observer selection and training

Eight observers assessed US, CBCT, and CBCT-US fusion image quality in this study. Observers were divided into 2 cohorts:

1. Experienced (3 observers): One clinician (IW) and 2 medical physicists (TOS and SM) with previous experience in interpreting both US and CBCT images.
2. New-to-US (5 observers): Clinicians, radiographers, and medical physicists who may have had experience in CBCT image analysis but had no prior training in interpreting US images.

Before performing the analysis, all 8 observers participated in a 1-hour training session (using the training dataset composed of 17 CBCT, US, and CBCT-US fusion images) designed to (1) teach observers how to use the MATLAB software application; (2) give observers experience in interpreting and contouring US, CBCT, and CBCT-US fusion images of the uterus; and (3) establish a consensus for rating contour confidence.

### Contour agreement metrics

The two geometric measures used to assess contour agreement were the Dice similarity coefficient (DSC)^27^ and the mean contour-to-contour distance (MCCD). For 2 contours X and Y, the DSC was calculated as (2|X∩Y|)/(|X|+|Y|), with 0 and 1 representing no overlap and perfect overlap, respectively, and the MCCD was defined as

(1) $$MCCD\;=\;\frac{1}{n}\sum_{i=1}^{n}\left|\left|X_{i}-Y_{i}\right|\right|$$

where n is the number of points comprising contour X.^28^

### Generation of the gold standard contour

For every image analyzed, a single contour was generated by combining the 3 contours from each of the observers in the Experienced cohort using Simultaneous Truth and Performance Level Estimation (STAPLE).^29^ The gold standard contour generated by the STAPLE method is the contour that optimizes the sensitivity and specificity of all of the contours from each individual observer. Because 40 CBCT, 40 US, and 40 CBCT-US fusion images were analyzed, this resulted in the generation of 120 gold standard contours. The contour agreement among the remaining 5 contours from the New-to-US cohort and the gold standard STAPLE contour was measured for each of the 120 images.

### Observer ratings

Observers were required to rate how confident they were that their contours reflected the true uterine boundary on a 10-point scale. Observers were asked to consider all factors potentially influencing their rating, including the visibility of the uterine boundary, the clarity of the bladder wall and bowel gas, the regularity of the uterine shape, and the position of anatomic landmarks with respect to the planning target volume. All observers had practice in using these factors to inform their rating during the 1-hour training session.

### Statistical analyses

The median and interquartile range (IQR) DSC, and MCCD between each of the 5 uterine contours in the New-to-US cohort and the gold standard STAPLE contour were reported for CBCT, US, and CBCT-US fusion images. Pairwise Wilcoxon signed-rank tests^30^ with Bonferroni correction were used to determine whether there were differences in the DSC and MCCD between imaging methods (US, CBCT, and CBCT-US fusion). The statistical analysis described was repeated for comparing the observer contour confidence ratings from all 8 observers from both the Experienced and New-to-US cohorts in CBCT, US, and CBCT-US fused images.

The interobserver contour agreement and contour confidence was also evaluated in the Experienced cohort to assess the influence of observer training on the results. A pairwise analysis was used to calculate the median and IQR of the DSC, MCCD, and confidence ratings of the contours from these 3 observers. Differences among US, CBCT, and CBCT-US fusion images were detected using a pairwise Wilcoxon signed-rank test with Bonferroni correction.

Spearman's rank order correlation coefficient (R_s_) and the corresponding *P* value were reported to assess the direction, strength, and significance of the association between the bladder area and the median contour confidence rating from all 8 observers for independent CBCT and US images.

## Results

The median (IQR) DSC and MCCD between observer contours from the New-to-US cohort and the gold standard STAPLE contours are shown in Table 1 for CBCT, US, and CBCT-US fusion images. The DSC between observer contours on CBCT-US fusion images was significantly greater than that between observer contours on CBCT (*P* = .002) and US images (*P* = 7.5e^−4^). The MCCD between observer contours on CBCT-US fusion images was statistically significantly lower than that between CBCT images (*P* = 3.4e^−20^) and US images (*P* = 3.6e^−24^). The MCCD was statistically significantly lower on CBCT images than on US images (*P* = 6.5e^−4^).

**Table 1:** Interobserver contour agreement and observer contour confidence results

	DSC	MCCD (mm)	Observer rating
CBCT	0.82 (0.14)	1.63 (0.74)∗	5 (4)
US	0.81 (0.17)	1.75 (1.15)	7 (2.75)∗
CBCT-US fusion	0.84 (0.11)†	1.26 (0.23)†	7 (2)†

*Abbreviations:* CBCT = cone beam computed tomography; DSC = Dice similarity coefficient; MCCD = mean contour-to-contour distance; US = ultrasound.

Median and interquartile range results for (columns 1 and 2) the DSC and MCCD between the gold standard contour and the 5 contours from the New-to-US cohort and (column 3) observer ratings of contour confidence (1 = extremely unconfident and 10 = extremely confident) from all 8 observers. Symbols indicate significant differences (*P* < .005) between imaging modalities within a column.

∗ Significant difference between CBCT and US.

† CBCT-US fusion is significantly different from both CBCT and US.

Median (IQR) contour confidence ratings were 5 (4), 7 (2.75), and 7 (2) for CBCT, US, and CBCT-US fusion images, respectively. The results from the Wilcoxon signed-rank tests revealed that (1) observers were significantly more confident in their contours drawn on CBCT-US fusion images compared with CBCT and US (*P* values of 2.7e^−28^ and 0.005, respectively), and (2) observers were significantly more confident in their contours drawn on US than on CBCT (*P* = 4.5e^−14^).

The median (IQR) DSC, MCCD, and observer ratings from the pairwise analysis of the Experienced cohort are shown in Table 2 for CBCT, US, and CBCT-US fusion images. There was no significant difference between the DSC on US and CBCT (*P* = 0.97), but the DSC between contours on the CBCT-US fusion was significantly higher than that for both CBCT and US (*P*-values of 5.8e^−4^ and 4.2e^−4^, respectively). All MCCD values and confidence ratings were significantly different between experienced observers on CBCT, US, and CBCT-US fusion images (*P* < 5e^−5^), with the CBCT-US fusion consistently outperforming CBCT and US.

**Table 2:** Pairwise interobserver contour agreement and confidence from Experienced observer cohort (3 observers)

	DSC	MCCD (mm)	Observer rating
CBCT	0.82 (0.13)	2.94 (1.58)	4 (4)
US	0.81 (0.14)	2.25 (2.67)∗	7 (3)∗
CBCT-US fusion	0.87 (0.07)†	1.63 (0.63)†	8 (2)†

*Abbreviations:* CBCT = cone beam computed tomography; DSC = Dice similarity coefficient; MCCD = mean contour-to-contour distance; US = ultrasound.

Median and interquartile range results from the pairwise analysis from the 3 observers in the Experienced cohort for (column 1) the DSC, (column 2) the MCCD, and (column 3) observer ratings of contour confidence (1 = extremely unconfident and 10 = extremely confident). Symbols indicate significant differences (*P* < .005) between imaging modalities within a column.

∗ Significant difference between CBCT and US.

† CBCT-US fusion is significantly different from both CBCT and US.

Spearman's correlation coefficient (R_s_) between bladder area and median contour confidence was –0.12 (*P* = 0.83) and 0.37 (*P* = 0.02) for CBCT and US images, respectively.

## Discussion

This study has demonstrated the superiority of combined US-CBCT imaging over either single modality for in-room target localization of the uterus during RT for cervix cancer. We have also shown that US alone is comparable to CBCT as a sole modality.

### CBCT versus US

There was no statistically significant difference in DSC between the two modalities; CBCT had a statistically significantly smaller MCCD than US; and observers were more confident in their US-based contours than in their CBCT-based contours. This indicates that, in general, CBCT and US have similar image qualities when used to visualize the uterus before RT in patients with cervix cancer.

**Fig. 1:**
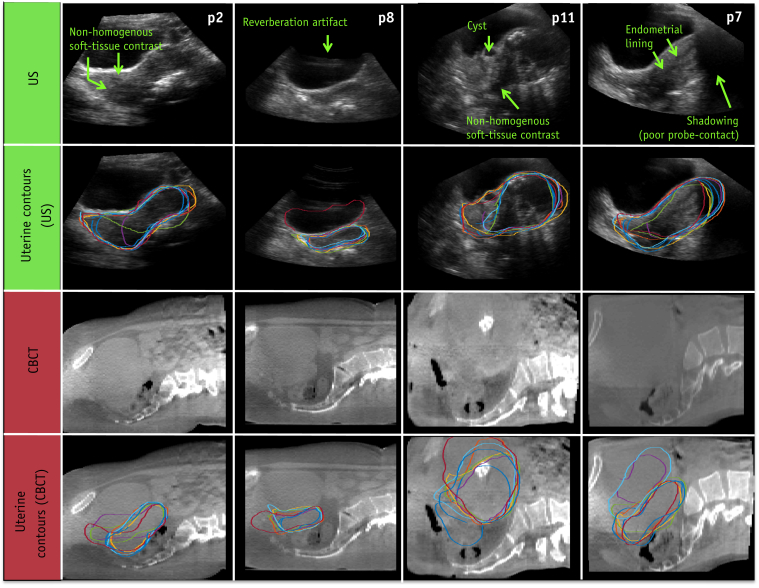
Examples of ultrasound (US) imaging features (row 1) that caused contouring disagreement (row 2) in patients 2, 8, 11, and 7 (p2-p11). Such features include nonhomogenous soft-tissue contrast in the uterus, internal structures such as cysts and the endometrial lining, and US artifacts such as shadowing and reverberations. Corresponding cone beam computed tomography (CBCT) and CBCT-based contours shown for reference in rows 3 and 4. The US and CBCT images are displayed on different scales (pixel sizes of [0.58, 0.58] mm and [2.5, 1] mm, respectively).

The reason why the higher observer ratings on US did not correspond with an improvement in interobserver contouring agreement in the New-to-US cohort is unclear, but this could be due to the limited amount of observer experience in interpreting US (1 hour) compared with CBCT (routine clinical use). This is corroborated by the analyses performed on the Experienced cohort, where the improved observer confidence rating in US image quality did correspond to a significantly improved MCCD compared to CBCT. It is well known that there is a steep learning curve in US image interpretation, partially because observers cannot rely on the body habitus or skeleton to orient themselves with the internal anatomy. Furthermore, inspection of the data also revealed that the observers in the New-to-US cohort sometimes misinterpreted the nonhomogeneous soft-tissue contrast, uterine substructures (e.g. cysts or the endometrial lining), or US artifacts as the uterine boundary; errors which could potentially be overcome with more training. Examples of such US artifacts are shown side by side with the corresponding CBCT with and without observer contours in Figure 1. Even with these challenges, interobserver contouring agreement on US in the New-to-US observer cohort matched that of CBCT after only a 1-hour training module.

In this work, potential geometric mismatches between US and CBCT were not explicitly accounted for. Factors such as probe pressure, gas movement, bulk, and patient motion could cause positional discrepancies between the apparent position of the uterus on the two modalities. However, preliminary measurements from this study did not detect a spatial mismatch: The median (IQR) DSC between the CBCT and US gold standard STAPLE contours was 0.78 (0.09), which is similar to the interobserver contouring variability of the uterus measured in previous work (median [IQR] DSC of 0.78 [0.11]).^31^

**Fig. 2:**
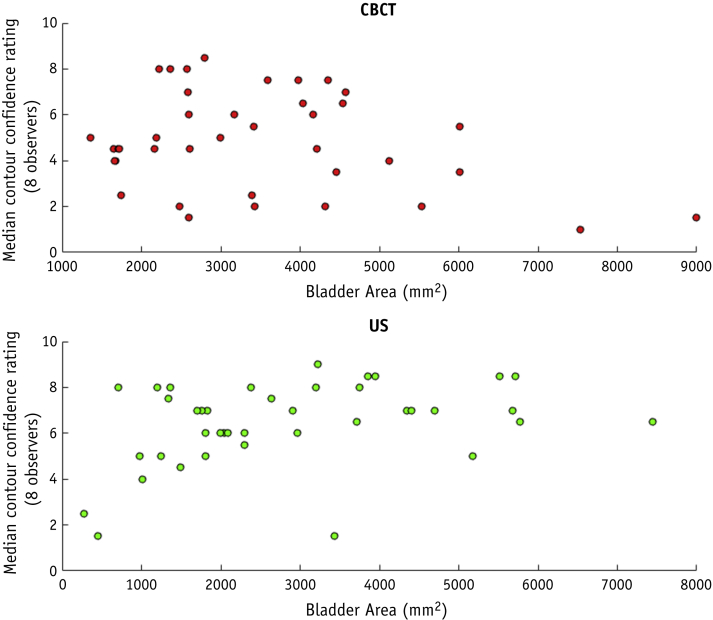
Scatter plots demonstrating the relationship between bladder size (measured as the area of the bladder on a central sagittal slice) and the median observer contour confidence rating for cone beam computed tomography images (top) and ultrasound images (bottom).

Because the bladder is situated anteriorly to the uterus, its volume can influence US image quality: A full bladder can provide an acoustic window to the gynecologic anatomy because urine has a low acoustic attenuation.^32^, ^33^ Although the correlation coefficient between bladder area and contour confidence was relatively low (0.37), it was statistically significant and in the expected direction, indicating that observer contour confidence increases with increasing bladder size. Aside from bladder size, other factors such as body mass index, age, and the presence of gas in the acoustic path could also affect US image quality, which may explain why the relationship between bladder size and US image quality was only weakly detected in this study. In comparison, a lesser association was expected between bladder size and CBCT image quality, which was reflected by the low, statistically insignificant R_s_ value of –0.12 between bladder size and contour confidence ratings on CBCT. The relationship between bladder size and median contour confidence ratings is displayed in Figure 2 for CBCT and US images.

In cases in which the uterus is clearly visualized on US, US could potentially replace CBCT for plan verification and plan of the day adaptive treatment for patients with cervix cancer. US could also be used for image guidance in centers without CBCT imaging systems (i.e., in low to middle income countries) or to reduce concomitant imaging dose from frequent image guidance.

### CBCT-US fusion

**Fig. 3:**
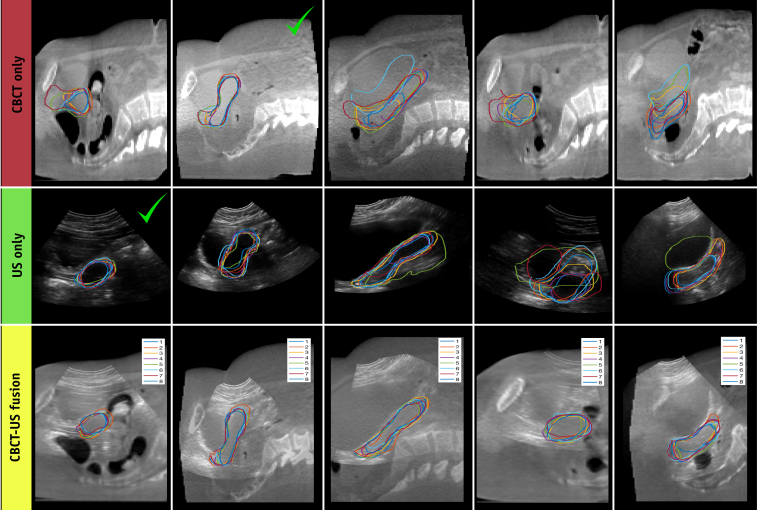
Comparison of interobserver contour agreement on cone beam computed tomography (CBCT) (pixel size [1, 1] mm), ultrasound (US) (pixel size [0.58, 0.58] mm), and CBCT-US fusion images (pixel size [0.58, 0.58] mm). Each observer's contour is denoted by the color given in the legend shown in the bottom row (Experienced observers = 1-3, New-to-US observers = 4-8). Columns 1-2: examples where the benefit of CBCT-US-fusion was mainly due to the increased probability of acquiring an excellent image from one modality (denoted by the green check marks). Columns 3-5: examples where CBCT and US images provided complementary information, which is why the interobserver agreement is relatively poor on both modalities individually but good on the CBCT-US fusion image. (A color version of this figure is available at https://doi.org/10.1016/j.ijrobp.2019.03.003.)

According to all of the metrics considered in this study (DSC, MCCD, and observer ratings), CBCT-US fusion images enabled observers to localize the uterus with more precision and confidence than either modality on its own. In some cases, US provided better visualization of the uterus, and in others CBCT did—as shown in Table 3, which compares the median DSC and MCCD values for each individual time point. Therefore, acquiring images from both modalities increases the probability of obtaining sufficient image quality to identify the uterus, which is a reason why the CBCT-US fusion outperformed US and CBCT alone (Figure 3, columns 1-2).

**Table 3:** Highest level contour agreement categorized by imaging method

	DSC	MCCD
CBCT	11 (27.5%)	5 (12.5%)
US	13 (32.5%)	5 (12.5%)
CBCT-US fusion	16 (40%)	30 (75%)

*Abbreviations:* CBCT = cone beam computed tomography; DSC = Dice similarity coefficient; MCCD = mean contour-to-contour distance; US = ultrasound.

Frequency that each modality (CBCT, US, and CBCT-US fusion) had the highest median DSC (column 1) and lowest median MCCD (column 2) in absolute cases and as a percentage of total time points (n = 40).

However, the dominant benefit of combing CBCT and US images lies in the fact that they provide complementary information and are most powerful when used together. This is clearly demonstrated by the MCCD, which was lowest in the CBCT-US fusion images 75% of the time compared with either individual modality (Table 3). The last column of Figure 3 exemplifies the complementary nature of CBCT and US; only the superoinferior extent of the uterus is visible on CBCT, whereas only the anteroposterior extent of the uterus is visible on US. Thus, the CBCT-US fusion enabled observers to combine the information from both modalities to determine the uterine boundary in all directions much more precisely. Columns 3 and 4 of Figure 3 further demonstrate the ability of observers to improve contouring precision on CBCT-US fused images, even when the uterine boundary on both modalities individually is unclear.

### Rationale for contouring in 2 dimensions rather than 3 dimensions

It has been established that uterine motion occurs predominantly in the superoinferior and anteroposterior anatomical directions,^5^, ^34^ and as such, this motion may be fully captured by 2-dimensional imaging along a central sagittal plane. Because this plane is also the most informative part of the 3D dataset, both in terms of establishing uterine position and for its information content for defining the uterine boundary by US, it has been used to manually initialize automated 3D uterine segmentation in Clarity's Assisted Gyne Segmentation algorithm.^31^ Because such automated segmentation algorithms would need to be used in an adaptive RT workflow to minimize contouring burden, 2-dimensional manual contour agreement on the central sagittal slice would be representative of uterine localization accuracy achievable in the clinic.

### Feasibility of integrating US into the clinical workflow

Integrating US into the RT workflow was easily achieved using the Clarity system, which enables the spatial registration of US images to treatment room coordinates via infrared probe-tracking technology. This made fusion with CBCT and CT trivial because all images were referenced to the same coordinate system. The small size of the system enabled us to leave it in the RT treatment room when not in use, making it easily accessible when needed. Daily quality assurance time is around 5 minutes. US acquisition and registration was easily incorporated into a standard treatment time slot (10 minutes).

Because uterine localization can be achieved using US with little impact on hospital resources, the benefits of daily US imaging for patients with cervical cancer may outweigh the cost of additional equipment. However, because US is not appropriate for determining the position of elective or involved nodal targets, we propose it either as an adjunct to other imaging modalities such as CBCT, or as a convenient and noninvasive soft-tissue verification method on days when CBCT imaging is not routinely performed.

### Future directions

Concern with regard to probe pressure effects and geometric accuracy of US images can be explored using CT or magnetic resonance imaging as the gold standard imaging modality. The development of a US training program for users and semiautomated segmentation could improve speed and accuracy of US-based verification. It also remains to be determined if patient and tumor characteristics pretreatment can predict the best imaging modality to use. Future work could include modifying the software program such that it records the time it takes for observers to identify the uterus on CBCT, US, and CBCT-US fusion images to see whether fusion improves observer speed as well as contour agreement.

## Conclusions

Target localization for cervix cancer RT is similar for CBCT and US. A weak association between bladder size and contour confidence on US images was observed, suggesting that larger bladder sizes may improve the quality of US images of the uterus. The combination of CBCT and US further improves the precision of target localization. Initial findings indicate that in cases in which the uterus is clearly visualized on US, US can be used as an alternative to CBCT for uterine localization, with the benefit of a reduction in radiation exposure and improved cost-effectiveness. Combined CBCT-US imaging could be used to implement adaptive planning strategies for cervix cancer RT.
